# A Multilevel Model of Teachers’ Job Performance: Understanding the Effects of Trait Emotional Intelligence, Job Satisfaction, and Organizational Trust

**DOI:** 10.3389/fpsyg.2018.02420

**Published:** 2018-11-30

**Authors:** Mingwei Li, Pablo Alejandro Pérez-Díaz, Yaqing Mao, K. V. Petrides

**Affiliations:** ^1^Faculty of Education, Beijing Normal University, Beijing, China; ^2^London Psychometric Laboratory, University College London, London, United Kingdom; ^3^Department of Clinical, Educational and Health Psychology, University College London, London, United Kingdom; ^4^Institute of Psychology, Southern University of Chile, Puerto Montt, Chile

**Keywords:** trait EI, job performance, job satisfaction, organizational trust, school, mediation, moderation, TEIQue

## Abstract

Research on the role of trait emotional intelligence (trait EI; Petrides, 2001) relating to teaching performance has emerged as an important topic. The present study proposes a multilevel model of teachers’ trait EI in relation to their job performance, which simultaneously addresses the mediating role of job satisfaction and the influences of school-level factors (i.e., organizational trust and principals’ trait EI). Results from a sample of 881 teachers and 37 principals in Chinese primary schools showed that job satisfaction partially mediated the positive relationship between teachers’ trait EI and their job performance. In addition, the findings demonstrated a cross-level moderated mediating effect, with the indirect effect of teachers’ trait EI on job performance (via job satisfaction) becoming stronger for teachers working in schools with lower levels of organizational trust. The hypothesized role of principals’ trait EI on teachers’ job performance was not supported. The theoretical and practical implications of these findings are discussed.

## Introduction

Teaching is generally recognized as one of the most important and challenging occupations in contemporary society (Vesely et al., 2013). These professionals are regarded to be responsible for their students’ academic achievement as well as social and emotional development (Elias and Arnold, 2006). Given the heavy demands and expectations in terms of students’ development, teachers’ job performance, which is tied to students’ outcomes (Hwang et al., 2017), is of crucial concern for a variety of stakeholders, including principals, parents, policymakers, and society at large (Alrajhi et al., 2017).

In recent years, a growing body of literature has highlighted the importance of emotional intelligence (EI) as a predictor for job performance, generally arguing that employees with higher levels of EI are likely to perform better (O’Boyle et al., 2011). In school settings, teachers are dependent on their interactions with many other school members to achieve their teaching goals (cf. Van Maele and Van Houtte, 2012). Teaching itself is also a form of emotional practice: emotion is at the heart of the teaching job (Hargreaves, 1998). It is, therefore, insufficient for school teachers to have only academic knowledge and instructional skills (Hosotani and Imai-Matsumura, 2011). There has been an increasing call for a greater focus on teachers’ EI to enhance performance (e.g., Collie et al., 2012; Corcoran and Tormey, 2012). However, research directly addressing the EI-job performance relationship among teachers is relatively scarce. Accordingly, the first goal of the present study is to investigate the association between trait EI and job performance in the teaching profession.

The existing literature concerning teachers’ trait EI has mainly focused on its effects on attitudinal outcomes, such as job satisfaction, burnout, and organizational commitment, among others (e.g., Chan, 2006; Wong et al., 2010; Anari, 2012). These variables may act as mediators in the relationship between teachers’ trait EI and job performance, building on previous claims that attitudes toward the job influence behaviors (Ajzen, 1987; Riketta, 2008). Among these variables, the current study focuses on the role of job satisfaction because the research on the relationship between satisfaction and performance is one of the most prominent in the literature. A number of studies have indicated that the focal attitude about one’s job (job satisfaction), is closely associated with job performance (e.g., Judge et al., 2001). Meanwhile, trait EI (Petrides, 2001) has been proposed as an important determinant of job satisfaction: employees with high trait EI are likely to have higher levels of job satisfaction than their low trait EI peers (e.g., Petrides and Furnham, 2006). Thus, it seems that job satisfaction could mediate the effect of teachers’ trait EI on their job performance.

In the occupational context, besides individual resources, leader and organizational resources have been shown to have strong associations with employees’ attitudinal and behavioral outcomes (Wayne et al., 1997). Thus, in addition to examining the above relationships at the individual level, the current research extends to the investigation of multilevel effects. First, current theories and findings indicate that the trait EI of leaders has a positive effect on followers’ job performance (e.g., Wong and Law, 2002; Miao et al., 2018). However, scholars in the educational context have rarely considered the role of principals’ trait EI when discussing teachers’ job performance. Second, researchers have noted that the effects of employees’ EI may depend on organizational contextual factors (e.g., Côté, 2014), which serve as boundary conditions that moderate the extent to which EI promotes work outcomes. Organizational trust is a key characteristic of contextual resources, which has been proved to influence teachers’ job outcomes (e.g., Van Maele and Van Houtte, 2012; Liu et al., 2016). However, existing studies on the effects of teachers’ EI are confined to the individual level and have resulted in a limited understanding of the complex ways in which it combines with organizational factors to influence job outcomes. We anticipated that principals’ trait EI would influence teachers’ job performance. Moreover, the relationship between teachers’ trait EI and job outcomes may be different in schools with different levels of organizational trust.

Although there has been increased recognition of the importance of trait EI in teachers’ work, research is only at the beginning stage. The present study is intended to contribute to the existing literature in several ways. First, our research focuses on the effect of teachers’ trait EI on their job performance. Although the link between teachers’ trait EI and some attitudinal outcomes has been studied, the evidence of its impact on behavioral outcomes is quite limited in the teaching profession. Second, the study seeks to examine the mediating role of teachers’ job satisfaction on the expected relationship between teachers’ trait EI and job performance. This could advance our understanding of the process by which teachers’ trait EI affects their job performance. Third, to overcome the limitation of focusing only at the individual level, we develop a multilevel model in which the predictive effect of principals’ trait EI and the moderating role of organizational trust are tested at the school level. In this way, the influence of principals’ trait EI and the interactive effect between teachers’ trait EI and organizational trust at the school level could be revealed in order to enable a clearer illustration of the mechanisms behind these relationships.

## Literature Review

### Trait EI and Job Performance

Trait EI (or trait emotional self-efficacy) is conceptualized as a constellation of emotional perceptions assessed through questionnaires and rating scales (Petrides et al., 2007b). Although being cognitively intelligent is still considered as an important attribute (Schmidt et al., 2008), EI has been increasingly regarded as a vital predictor of adaptation and success in the workplace (e.g., Wong and Law, 2002; Petrides and Furnham, 2006; Sy et al., 2006).

Job performance, defined as the set of behaviors an individual performs toward achieving the goals of an organization (Motowidlo and Van Scotter, 1994), is the focal outcome in the workplace. Studies utilizing both trait and ability EI measures have reported converging results in terms of their effects on job performance. For example, a large-scale meta-analysis conducted by O’Boyle et al. (2011) showed that employees’ EI exhibited substantial relative importance even when the Five Factor Model (FFM) and cognitive intelligence were controlled for. The different measures of EI showed corrected correlations with job performance ranging from 0.24 to 0.30. The ability model of EI using maximum-performance measurements was more closely related to cognitive intelligence, resulting in lower incremental validity compared to the trait model in terms of predicting job performance (O’Boyle et al., 2011). Thus, while both ability EI and trait EI are relevant to job performance, the focus of the current study is on the latter.

In the educational context, schools are recognized as key organizations for developing the academic, social, and emotional competence of students (Roeser et al., 2000). Teachers’ job performance can be defined as the actions they perform in schools in order to achieve educational goals (Hwang et al., 2017). The job role of the teacher is highly emotion-driven (Hargreaves, 1998) and largely dependent on interactions with other members of the school community (Van Maele and Van Houtte, 2012), thus highlighting the role of teachers’ trait EI in generating good teaching performance (Alrajhi et al., 2017). Teachers’ trait EI could be beneficial to their job performance in two ways. Intrapersonally, better awareness of their own emotions can help teachers to build more confidence and control over their teaching tasks, which, in turn, enables better performance. Interpersonally, by allowing them to understand and manage the emotions of others (e.g., colleagues and students), teachers’ trait EI may contribute to positive social interactions and, thus, more effective teaching. Results from a limited number of studies have shown a positive correlation between teachers’ trait EI and job performance, indicating that teachers with high trait EI are likely to perform better. For example, by adapting several previous trait EI measurements, Myint and Aung (2016) categorized teachers’ trait EI into four factors: utilization of emotion, optimism/mood regulation, expression/appraisal of emotion, and emotional resilience. Based on a sample of 1,006 school teachers, their results revealed that 8.1% of the variance in teachers’ job performance was explained by “optimism/mood regulation” and “expression/appraisal of emotion”. Similarly, Naqvi et al. (2016) conducted a study among 3,168 teachers using the TEIQue-SF (Petrides, 2009), discovering that teachers’ trait EI had a positive relationship with their job performance (*r* = 0.11, *p* < 0.01).

**Hypothesis 1:** Teachers’ trait EI positively predicts their job performance.

### The Mediating Role of Job Satisfaction

The attitudinal approach to defining job satisfaction, which concerns the evaluative judgments people make about their jobs (Weiss, 2002), is prevalent in the literature. Numerous meta-analyses (e.g., Petty et al., 1984; Judge et al., 2001) have established a robust correlation between job satisfaction and job performance. According to the human relations theory, satisfaction causes performance (cf. Petty et al., 1984): employees who are more satisfied with their work tend to perform better than their less satisfied peers in the workplace.

Teachers’ job satisfaction can be conceptualized as teachers’ affective reactions to their work (Skaalvik and Skaalvik, 2011). Due to its predictive effect on performance-related variables, research in several different cultures indicates that teachers’ job satisfaction is of central interest in the educational literature (e.g., Bogler, 2001; Crossman and Harris, 2006). Teachers who are satisfied with their job are more involved in it (Weiqi, 2007), more likely to take on extra-role activities (Somech and Drach-Zahavy, 2000), and less likely to leave the teaching profession (Skaalvik and Skaalvik, 2011). Moreover, teachers’ job satisfaction may contribute to students’ optimal development and overall school effectiveness, which are indicators of teaching performance (e.g., Ostroff, 1992; Caprara et al., 2006).

In an eye-tracking study, Lea et al. (2018) found that higher trait EI was associated with more attention to positive emotional stimuli, relative to negative and neutral stimuli. Such an attentional preference may be one way that trait EI affords protection from stressors, thus promoting job satisfaction in the workplace (Yin et al., 2013; Lea et al., 2018). Indeed, many studies have reported a positive relationship between employees’ trait EI and job satisfaction (e.g., Carmeli, 2003; Petrides and Furnham, 2006). In addition, Miao et al. (2017a,b) confirmed in two meta-analyses that trait EI predicts job satisfaction directly as well as incrementally beyond cognitive ability and the Big Five personality traits (see also Andrei et al., 2016).

Anari (2012), using an adaptation of Schutte et al.’s (1998) scale, found a positive correlation between trait EI and job satisfaction among high school teachers (*r* = 0.23, *p* < 0.05). Using the WLEIS (Wong and Law, 2002), Wong et al. (2010) reported a similar correlation between trait EI and job satisfaction among 3,866 teachers in Hong Kong (*r* = 0.30, *p* < 0.01). Also using the WLEIS, Yin et al. (2013) conducted a survey among 1,281 Chinese school teachers and SEM results indicated that a second-order trait EI factor was a significant positive predictor of teaching satisfaction (estimate = 0.30, *p* < 0.01).

Based on the relationships between teachers’ trait EI, job satisfaction, and job performance, we expect that job satisfaction will act as a mediator of the impact of teachers’ trait EI on job performance. Indeed, considerable research has shown that personality traits can influence job performance through the mediating effects of attitudinal processes (e.g., Barrick et al., 2002; Mount et al., 2006). Moreover, there is also evidence supporting the specific mediating role of job satisfaction in the relationship between employees’ trait EI and performance-related variables. For example, using the WLEIS (Wong and Law, 2002; Brunetto et al., 2012) found that employees’ trait EI positively predicted job satisfaction, which, in turn, promoted employee engagement. Based on this literature, we propose in the current study that job satisfaction acts as a mediator of the relationship between teachers’ trait EI and job performance.

**Hypothesis 2:** Job satisfaction mediates the relationship between teachers’ trait EI and job performance.

### The Moderating Role of Organizational Trust

Research has demonstrated that employees’ trait EI is related to job attitudes and behaviors, however, its impact may vary across different organizations. According to the principles of trait activation theory (Tett and Burnett, 2003), personality traits require trait-relevant situations for expression. Specifically, a trait is more likely to be activated in amenable situations signaling to individuals that expressing it is both important and appropriate (Tett and Burnett, 2003). Accordingly, the relationship between employees’ trait EI and job outcomes may differ depending on contextual factors. Indeed, emerging literature relating employees’ EI to job outcomes demonstrates the importance of contextual factors, which should be considered as important moderators (e.g., Cherniss, 2010; Côté, 2014).

One key contextual factor within all organizations is trust (Dirks and Ferrin, 2001). Organizational trust is defined as “the willingness of a party to be vulnerable to the actions of another party” (Mayer et al., 1995, p. 712). In the educational literature, Hoy and Tschannen-Moran (1999) defined trust as “a teacher’s willingness to be vulnerable to another party based on the confidence that the latter is benevolent, reliable, competent, honest, and open” (p. 189). In the current study, we operationalized trust at the organizational level, rather than the individual level. This focus on school-level trust is consistent with previous research showing that individual teachers’ perceptions of trust can merge to form a collective state at the school level (e.g., Forsyth et al., 2011; Van Maele and Van Houtte, 2012). Teachers in the same school are supervised by the same leader(s) and work with the same group of colleagues, thus involved in similar social interactions. Based on the experience of these shared social interactions, teachers within the same school are likely to share similar perceptions of organizational trust (Salancik and Pfeffer, 1978). In the current study, organizational trust was conceptualized as teachers’ perceptions of other school members’ trustworthiness (Van Maele and Van Houtte, 2012).

Across organizational settings, employees feel safer, more positive, and less insecure when they believe that their leaders and peers are trustworthy (Dirks and Ferrin, 2002). In contrast, low levels of trust lead to self-estrangement, powerlessness, and conflict (Hoy and Tschannen-Moran, 1999). Studies have indicated that trust promotes employee satisfaction in organization settings (e.g., Shockley-Zalabak et al., 2000; Dirks and Ferrin, 2001). However, in the educational literature, research on the relationship between teachers’ trust relationships and their job satisfaction is limited. One important study was conducted by Van Maele and Van Houtte (2012). Using multilevel analyses, they related trust, at the level of both the individual teacher and the collective faculty, to teachers’ job satisfaction. The results confirmed a positive effect of individual perceptions of trust on teachers’ job satisfaction and found that only 2.72% of the variance in teachers’ job satisfaction was situated at the school level. Despite the small number of studies focusing directly on the role of trust in generating job satisfaction, its importance can be interpreted from other perspectives. In particular, the social capital approach has been frequently used in the educational literature to investigate trust relationships in school settings (cf. Van Maele and Van Houtte, 2012). Trust among teachers is a component of the relational dimension of social capital in schools (Nahapiet and Ghoshal, 2000), which indicates the quality of social relationships. According to Dinham and Scott (1998), school-based positive relationships are regarded as an important source of teachers’ job satisfaction. For example, by using SEM analyses, Skaalvik and Skaalvik (2011) found that teachers’ positive relationships with principals and colleagues were predictive of job satisfaction through the feeling of belonging. In light of the foregoing, it is reasonable to expect that teachers in schools with higher levels of organizational trust will experience higher levels of job satisfaction.

Organizational trust at the school level could influence the relationship between teachers’ trait EI and job satisfaction differently across different schools. A multilevel model was, therefore, developed to investigate possible cross-level moderation effects. Specifically, in line with the trait activation theory (Tett and Burnett, 2003), we propose that a low level of organizational trust increases the salience of trait-relevant cues and representative situations pertinent to trait EI. In such schools, teachers are required to skillfully interpret and regulate emotions internally and externally, as this helps them to overcome a climate of low trust and maintain job satisfaction. It follows that the positive relationship between teachers’ trait EI and job satisfaction may be strengthened in schools with low levels of organizational trust. Conversely, teachers in schools with high levels of organizational trust may be more easily satisfied, reducing the necessity of emotionally intelligent behaviors. Thus, a high level of organizational trust is likely to weaken the positive relationship between teachers’ trait EI and job satisfaction. Although we are not aware of any prior empirical research addressing the moderating effect of organizational trust in this relationship, the theoretical rationale above leads us to our third hypothesis:

**Hypothesis 3:** Organizational trust moderates the relationship between teachers’ trait EI and job satisfaction.

The arguments above form an integrated framework in which job satisfaction mediates the relationship between teachers’ trait EI and job performance, while organizational trust moderates the relationship between teachers’ trait EI and job satisfaction. Taken together, these hypotheses give rise to a multilevel moderated mediation model (Edwards and Lambert, 2007), in which the indirect effect of teachers’ trait EI on job performance through job satisfaction varies as a function of the cross-level moderator, viz, organizational trust. Hence, organizational trust, due to its moderating power on the relationship between teachers’ trait EI and job satisfaction, has the potential to moderate the indirect (i.e., mediated) effect of teachers’ trait EI on job performance via job satisfaction.

**Hypothesis 4:** Organizational trust moderates the mediating effect of job satisfaction in the relationship between teachers’ trait EI and job performance.

### The Role of Principals’ Trait EI

Although the impact of leaders’ EI on the work outcomes of their employees has been extensively studied (Prati et al., 2003; Kerr et al., 2006; Miao et al., 2016), the specific role of principals’ trait EI in the educational field is sparsely documented.

Leadership theories suggest that EI is crucial for leaders because of their interactions with employees. EI is typically linked to effective leadership styles (Palmer et al., 2001), which are positive predictors of followers’ work outcomes (Dvir et al., 2002). For example, Barling et al. (2000) found that managers’ trait EI significantly predicted three aspects of transformational leadership (i.e., idealized influence, inspirational motivation, and individualized consideration) based on multivariate analyses of covariance. Similarly, Schlechter and Strauss (2008) tested a SEM model in a manufacturing company finding that team-leaders’ EI was positively correlated with transformational leadership behaviors. Siegling et al. (2014), studied the trait EI of 128 managers using the TEIQue-SF (Petrides, 2009), and found that it was significantly higher than normative data would predict, suggesting that high trait EI is a common characteristic among leaders. In addition, Mikolajczak et al. (2012) discovered that managers with low trait EI had difficulties putting their emotions aside compared to their average and high trait EI peers based on a sample of over 200 managers.

The literature also supports the direct relationship between leaders’ EI and subordinates’ attitudinal and behavioral work outcomes. For example, Wong and Law (2002) conducted a survey among 146 middle-level administrators in the Hong Kong government and found that the EI of leaders was positively related to the job satisfaction and extra-role behaviors of their followers. Using the WLEIS (Wong and Law, 2002; Sy et al., 2006) collected data from 187 food service workers and their 62 managers and similarly found that managers’ EI was positively related to employees’ job satisfaction and job performance. Clarke and Mahadi (2017), using the EIS (Schutte et al., 1998), found that the trait EI of both managers and subordinates were positively associated with mutual recognition respect, which, in turn, predicted subordinates’ job satisfaction and affective commitment. Last, Miao et al. (2018) showed in a meta-analysis that leaders’ trait EI significantly predicted subordinates’ job performance (operationalized as task performance and organizational citizenship behavior).

The importance of EI for educational leadership in the school context has also been highlighted in the literature (Brinia et al., 2014). Existing studies have related leaders’ trait EI to teachers’ attitudinal outcomes. For example, Wong et al. (2010) studied the role of trait EI, using the WLEIS (Wong and Law, 2002), in a large sample of teachers and middle-level leaders in Hong Kong. They discovered that the trait EI of middle-level leaders had a significant impact on the job satisfaction of ordinary frontline teachers, even after controlling for the job satisfaction of leaders and the base trait EI of frontline teachers. However, empirical evidence concerning the relationship between principals’ trait EI and the focal behavioral outcome of teachers remains scarce.

**Hypothesis 5:** Principals’ trait EI positively predicts teachers’ job performance.

**FIGURE 1:**
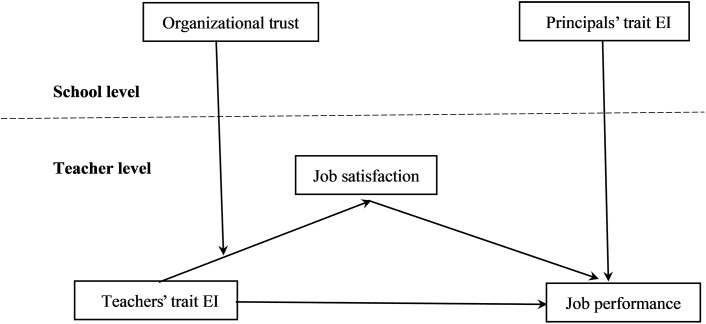
Overview of the hypothesized model.

To summarize, we have proposed that (a) teachers’ trait EI is positively associated with job performance via job satisfaction (Hypotheses 1 and 2); (b) teachers’ trait EI is more strongly related to job satisfaction and, in turn, to job performance when the level of organizational trust is low (Hypotheses 3 and 4); and (c) principals’ trait EI positively predicts teachers’ job performance (Hypothesis 5). The integrated model is outlined in Figure 1.

## Materials and Methods

### Participants

Thirty-seven principals (94.6% male) and 881 primary school teachers (73.3% female) were drawn from 37 public primary schools in the Hubei Province in mainland China. Eight schools were located in cities and the remainder in rural areas. A minimum of 40% of the teachers in each school participated in the study.

#### Teachers’ Demographics

Most teachers were in their early-to-middle adulthood (*Mage* = 37.57 years, *SDage* = 10.15, *Minimum* = 18, *Maximum* = 63). Teaching experience was substantial at *M* = 16.93 years and *SD* = 11.80. Teaching experience at the current school was also considerable (*M* = 9.77, *SD* = 9.53). Most teachers worked directly in the classroom (*fo* = 684, 78%), while a smaller percentage assumed leading positions, where mid-level leaders represented 17% of the sample (*fo* = 154), and school-level leaders 4% (*fo* = 34). Finally, 1% performed other functions in the schools. Regarding educational background, 58% (*fo* = 507) held a bachelor-degree, 36% (*fo* = 313) a college-degree and 7% (*fo* = 59) a high-school degree or below. Finally, the percentage of teachers with a Master degree or above was below 1%.

#### Principals’ Demographics

Most principals were in their mid-adulthood (*M* = 46.16 years, *SD* = 4.79). They had been leading their current schools for a minimum of 1 and a maximum of 14 years (*M* = 4.20 years, *SD* = 3.25, *Q1* = 2, *Q2* = 3, *Q3* = 6.5). On average, for the 37 schools that took part in the study, each principal accounted for 43 teachers (*SD* = 40.85). The large dispersion can be explained by the size of the schools, such that some had only a handful of teachers, while others had well over 200. Regarding educational background, 97% of the principals held either a college or a bachelor-degree. Noteworthy, none held postgraduate degrees, which reflects the fact that experience is more highly regarded than postgraduate qualifications in the Chinese system.

### Measures

#### Trait Emotional Intelligence

The Chinese adaptation of the Trait Emotional Intelligence Questionnaire-Short Form (TEIQue-SF; Petrides, 2009) was employed to assess teachers’ and principals’ trait EI. The 30-item short form was specifically designed as an efficient measure of global trait EI. A sample item is “I’m usually able to find ways to control my emotions when I want to.” For teachers, the level of analysis was the individual. For principals, trait EI was modeled as a top-down factor in our model and was analyzed at the school level. The internal consistency reliability of the scale was 0.86 and 0.87 for teachers and principals, respectively. The instrument has been extensively validated in Europe (Petrides et al., 2007a) and in China (Shao et al., 2013; Gökçen et al., 2014).

#### Job Performance

The Chinese version of job performance scale (Xingchun and Dajun, 2011) was adopted from the scale originally developed by Motowidlo and Van Scotter (1994). The 14-item instrument measures self-rated job performance. A sample item is “I work overtime to complete a task.” Teachers responded to the items on a 6-point Likert scale. Item responses were summed up and averaged to derive a total scale score, whose internal consistency reliability was 0.95. The scale has been widely used in previous studies with high reliability and validity (e.g., Kiker and Motowidlo, 1999; Hou et al., 2014).

#### Job Satisfaction

We measured job satisfaction using Zhi-yong and Zhi-hong’s (2012) 10-item scale. Items were responded to on a 5-point Likert scale. A sample item is “I am pleased with the welfare benefits provided by the school.” Item responses were summed up and averaged to derive a total scale score. Higher scores indicate higher levels of teacher job satisfaction. In our study, the internal consistency reliability was 0.93.

#### Organizational Trust

The Chinese adaptation (Jia et al., 2006) of the organizational trust scale (Robinson, 1996; Romano, 2003, Unpublished) was used. The 6-item instrument measures the extent to which teachers trust other members in their school. Items were responded to on a 5-point Likert scale. A sample item is “I believe my school will protect my interest.” Scale scores were derived by summing up and averaging the responses to the items. Higher scores indicate higher levels of organizational trust. In this study, the internal consistency reliability was 0.96. The instrument’s validity has been supported by previous research (Ying and Xi, 2014).

#### Control Variables

We also included several individual demographic characteristics in the analyses because these variables may potentially impact the relationships of interest (e.g., Chan, 2004; Petrides and Furnham, 2006; Ju et al., 2015). Specifically, gender, age, teaching tenure and educational background were added as control variables. However, gender, age, and educational background were not significantly related to the dependent variable in the current study and were, accordingly, removed.

### Procedure

Before conducting the survey, the researchers were granted permission from the Department of Education of the Hubei Province in China. Subsequently, invitation letters were sent to the principals and teachers of the sampled 37 schools. In the invitation letters, the nature, purpose, and method of the survey were clearly stated. Principals and teachers were asked to complete the questionnaires either in their offices or in conference rooms in their own schools. Both principals and teachers completed the TEIQue-SF, while teachers also completed the job performance, job satisfaction, and organizational trust questionnaires.

All participants were informed that their participation in the research was on a voluntary basis and their responses would be kept anonymous and confidential. Written informed consent was obtained from all participants in the study. The study protocol was approved by the Ethics Committee of Beijing Normal University, China.

### Aggregation of Organizational Trust

In our study, organizational trust was operationalized as an aggregate of individual responses at the school level. It has been proposed by researchers (e.g., Bliese, 2000) that there are three steps to determine the viability of aggregation: “sufficient within-group homogeneity, sufficient between-group heterogeneity, and that the group is naturally occurring” (cf. Wallace et al., 2016). In the current study, the grouping variable (i.e., schools) does occur naturally, thus, establishing sufficient within-group homogeneity and between-group heterogeneity were required to justify aggregation. The *r*_wg(j)_ statistic (James et al., 1993) was calculated as an indicator of agreement within schools. The median *r*_wg(j)_ across the schools was 0.90, ranging from 0.70 to 0.98, suggesting that in all schools, teachers shared common perceptions regarding organizational trust. Additional support for aggregating organizational trust at the school level was provided by interrater reliability indexes (intraclass correlation *ICC* [1] = 0.10 and reliability of team means *ICC* [2] = 0.63). Moreover, the between-groups variance was tested to indicate whether perceptions of organizational trust varied across different schools. The result was *F*(36,844) = 3.14, *p* < 0.001, which justified the use of the aggregate organizational trust score for the purposes of the school-level analyses.

### Strategy of Analysis

In the current study, teachers were nested within their schools, which forms a hierarchical structure. Furthermore, certain hypotheses (i.e., Hypotheses 3, 4, and 5) involved multilevel relationships between school-level variables and individual-level variables. Therefore, multilevel modeling was used to simultaneously estimate the hypothesized relationships using Mplus 7 (Muthén and Muthén, 2012). Before analysing cross-level effects, the variables were centered, according to the recommendations in Enders and Tofighi (2007). Specifically, Level 1 variables were group-centered to ensure that there was no conflation of the individual and school-level effects, in order to obtain an unbiased estimate. In addition, Level 2 variables were grand-centered to help with interpretations of the interaction effects.

## Results

### Preliminary Analyses

The distribution was analyzed taking the variable job performance as criterion with the original 890 collected observations. For accuracy, eight cases were removed from the analysis, as they were considered outliers according to normality analyses and Quantile-Quantile and Steam-Leaf plots. After removing these outliers, the following descriptive statistics were obtained: *Mjp* = 5.15, *SD* = 0.59, *C.I*. lower bound = 5.11, *C.I.* upper bound = 5.19, Skewness = -0.314 with *SE* = 0.082 and Kurtosis = -0.704 with *SE* = 0.165.

Table 1 shows the descriptive statistics and correlation matrix for the key variables in the study. Teachers’ trait EI was positively correlated with teachers’ job performance (*r* = 0.45, *p* < 0.01), job satisfaction (*r* = 0.30, *p* < 0.01) and teachers’ organizational trust (*r* = 0.26, *p* < 0.01). In addition, teachers’ job satisfaction was positively correlated with job performance (*r* = 0.44, *p* < 0.01) and organizational trust (*r* = 0.69, *p* < 0.01). Last, teachers’ organizational trust was positively correlated with teachers’ job performance (*r* = 0.34, *p* < 0.01).

**Table 1:** Descriptive statistics and correlations between variables.

Variables	*M*	*Maximum*	*Minimum*	*SD*	*1*	*2*	*3*	*4*
1. Teachers’ trait El	4.86	6.80	3.10	0.65				
2. Teachers’ job performance	5.15	6.00	3.64	0.58	0.45^∗∗^			
3. Teachers’ job satisfaction	3.78	5.00	1.10	0.78	0.30^∗∗^	0.44^∗∗^		
4. Teachers’ organizational trust	4.09	5.00	1.00	0.83	0.26^∗∗^	0.34^∗∗^	0.69^∗∗^	-
5. Teaching tenure	–	–	–	–	0.04	0.12^∗∗∗^	0.005	-0.08^∗^
6. Principals’ trait El	4.80	5.67	4.03	0.44				


*N = 881 for teachers. *N* = 37 for principals. Organizational trust values are for individual perceptions before aggregation at the school level. ^∗∗^*p* < 0.01, ^∗∗∗^*p* < 0.001.*

### Main Analyses

Before testing the multilevel model, we examined relationships at the individual level. We followed Preacher et al.’s (2010) guidelines and tested a path model, in which the indirect effect of teachers’ trait EI on job performance through job satisfaction was assessed, while the direct effect and the nesting of teachers within schools (i.e., the inclusion of random intercepts and slopes) were simultaneously specified. In addition, teaching tenure was included as a control variable with a fixed effect on job performance. This analysis allows incorporating Preacher and Hayes’s (2004) simultaneous estimation method of testing mediation effects, rather than relying on stepwise procedures as previously recommended by Baron and Kenny (1986). Moreover, it allows an estimation of the variability in the effects over Level 2 units (i.e., schools) in order to justify the need for investigating cross-level moderation effects (Bauer et al., 2006).

Results showed that teachers’ trait EI was positively related to job performance (γ = 0.33, *p* < 0.001), thus supporting Hypothesis 1. In addition, job satisfaction was positively related to job performance (γ = 0.24, *p* < 0.001), and teachers’ trait EI was positively related to job satisfaction (γ = 0.36, *p* < 0.001). To provide a test of the indirect effect (Hypothesis 2), we used a parametric bootstrap procedure to estimate a confidence interval (CI) around the indirect effect (Preacher et al., 2010). With 20,000 Monte Carlo replications, results showed that there was a positive indirect relationship between teachers’ trait EI and job performance via job satisfaction (indirect effect = 0.06, 95% bias-corrected bootstrap CI [0.018–0.105]). These results provided support for Hypothesis 2. Meanwhile, for the individual-level analysis, we also found significant random effects for teachers’ trait EI (*p* < 0.05), indicating that there could be school-level moderators which can explain this variability (Kenny et al., 2003). The goodness of fit statistics for this model with 23 degrees of freedom and *N* = 881 were: *-2LL* = 3060.74, *Akaike (AIC)* = 3106.74, *BIC* = 3216.63 and *saBIC* = 3143.58.

**FIGURE 2:**
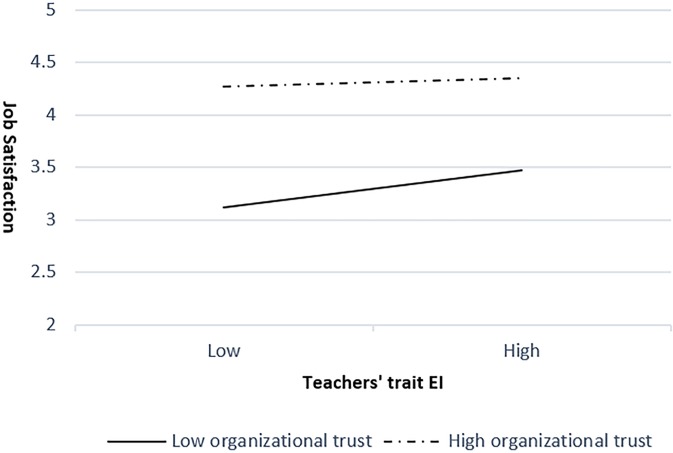
The interaction effect between teachers’ trait EI and organizational trust for job satisfaction.

At the school level, organizational trust was hypothesized to moderate the relationship between teachers’ trait EI and job satisfaction. To estimate the cross-level effect, we tested a model with teachers’ trait EI on job satisfaction, including a random slope from organizational trust as moderator. The multilevel modeling results indicated a negative effect of organizational trust on the random slope linking teachers’ trait EI and job satisfaction (γ = -0.64, *p* < 0.001), which represents a cross-level interaction. Therefore, Hypothesis 3 was borne out by the data, providing necessary initial support for the first stage of testing the moderated mediation model (Edwards and Lambert, 2007), proposed in Hypothesis 4. Following Aiken and West’s (1991) procedures, we plotted the interaction at higher (1 *SD* above the mean) and lower (1 *SD* below the mean) levels of organizational trust. As shown in Figure 2, the positive relationship between teachers’ trait EI and job satisfaction is stronger when organizational trust is low (solid line) than when it is high (dashed line). The goodness of fit statistics for this model with eight degrees of freedom and *N* = 881 were: *-2LL* = 1865.26, *Akaike (AIC)* = 1881.26, *BIC* = 1919.48 and *saBIC* = 1894.07.

To overcome limitations associated with a stepwise approach to testing a conceptual model in which there is both mediation and moderation, we independently examined our results utilizing an integrative approach. We followed the method outlined by Bauer et al. (2006) for determining the significance of conditional indirect effects in the context of multilevel regression by estimating the indirect effect of teachers’ trait EI on job performance via job satisfaction at higher (1 *SD* above the mean) and lower (1 *SD* below the mean) levels of organizational trust. The results revealed that the indirect effect of teachers’ trait EI on job performance via job satisfaction differed as a function of organizational trust. Specifically, the indirect effect of teachers’ trait EI was stronger when organizational trust was lower (estimate = 0.13, *SE* = 0.03, *p* < 0.001) and weaker when organizational trust was higher (estimate = 0.04, *SE* = 0.02, *p* < 0.05) in the schools, which was in line with Hypothesis 4. The goodness of fit statistics for this model with 33 degrees of freedom and *N* = 881 were: *-2LL* = 6392.90, *Akaike (AIC)* = 6458.91, *BIC* = 6616.57 and *saBIC* = 6511.77.

**FIGURE 3:**
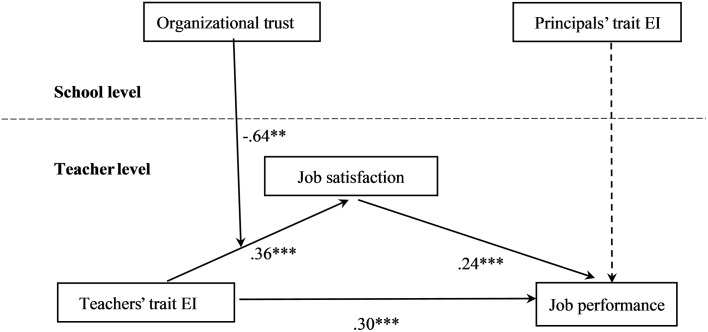
Multilevel moderated mediation model path coefficients. The control variable (teaching tenure) is not depicted in the figure. ^∗∗^*p* < 0.01, ^∗∗∗^*p* < 0.001.

Next, the predictive role of principals’ trait EI on teachers’ job performance was included in the model. As shown in Figure 3, all relationships in the proposed model were significant (*p* < 0.01) except the direct effect from principals’ trait EI (γ = -0.08, *p* > 0.05) on job performance. Thus, Hypothesis 5 was not borne out by the data. The goodness of fit statistics for the full model with 32 degrees of freedom and *N* = 881 were: -*2LL* = 6347.95, *Akaike (AIC)* = 6411.95, *BIC* = 6564.84 and *saBIC* = 6463.21. This last multilevel model was significantly better than the previous one, with a –2 Log-Likelihood change of 44.95 (Δ *df* = 1, *p* < 0.001).

Finally, we calculated Snijhders and Bosker’s (1999) overall pseudo *R*^2^ (*∼R*^2^) for the model, which is based on the proportional reduction of Level 1 and Level 2 errors due to the predictors in the model. The predictors accounted for 15% of the total variance in job performance, suggesting that teachers’ trait EI, job satisfaction and organizational trust in their schools were indeed important in predicting job performance.

## Discussion

### Theoretical Implications

Our results have several theoretical implications. First, they supported the positive relationship between teachers’ trait EI and job performance. The finding is consistent with prior research indicating the positive effect of employees’ trait EI on job performance within and beyond the teaching profession (e.g., Petrides and Furnham, 2006; Myint and Aung, 2016). According to O’Boyle et al.’s (2011) meta-analysis in the industrial psychology literature, there is a positive link between employees’ EI and job performance, ranging from 0.24 to 0.30. In our study, the correlation coefficient between teachers’ trait EI and job performance was quite higher at *r* = 0.45. As has been noted in previous research, EI is likely to be more important for jobs involving frequent social interactions or significant levels of stress (Cherniss, 2010). This high correlation coefficient might thus be a natural result of the intrinsic nature of the teaching job, which involves high levels of social interaction (e.g., with students and colleagues; Van Maele and Van Houtte, 2012) and serious stress-related challenges (Chan, 2006). “Understanding, negotiating and monitoring the intense emotionality” (Intrator, 2006) in themselves and others is a primary dimension of their work, which renders teachers’ trait EI central to their job performance.

Additionally, the current study found that job satisfaction partially mediated the relationship between teachers’ trait EI and job performance (indirect effect = 0.06). This effect helps us understand the process through which teachers’ trait EI improves job performance. Previous theories and studies have considered the direct relationships between trait EI, job satisfaction, and job performance among teachers (e.g., Skaalvik and Skaalvik, 2011; Yin et al., 2013). It has also been argued that personality traits can influence behaviors through the mediating effects of attitudinal processes (e.g., Barrick et al., 2002; Mount et al., 2006). However, few studies have examined job satisfaction as the mediator of the relationship between teachers’ trait EI and job performance. Our results suggest that teachers with higher trait EI perform better than those with lower trait EI, partially because they are more satisfied with their jobs, which, in turn, leads to better performance.

The current study is one of the first empirical investigations of the role of contextual factors in models of EI relating to job outcomes (Cherniss, 2010; Côté, 2014). By developing and testing a multilevel moderated mediation model, our study indicated that organizational trust at the school level serves as an important boundary condition for the effects of teachers’ trait EI (γ = -0.64). Specifically, in line with trait activation theory (Tett and Burnett, 2003), the findings supported our hypothesis that higher levels of organizational trust in schools render the trait EI of teachers less important in terms of predicting job outcomes. In contrast, when a lower level of organizational trust is present in a school, the importance of teachers’ trait EI is highlighted. This result is consistent with the reasoning of Dirks and Ferrin (2001), which established organizational trust as a prominent framing condition for understanding work outcomes, and also with previous research demonstrating the moderating role of trust climate in team settings (e.g., Brahm and Kunze, 2012).

In our study, teachers in schools with high levels of organizational trust experienced higher job satisfaction than those in schools with low levels of organizational trust (see Figure 2). This result is also in agreement with studies demonstrating the role of trust in school settings, especially in relation to job satisfaction (e.g., Van Maele and Van Houtte, 2012). Given that teachers in schools with higher levels of organizational trust already have higher job satisfaction than their peers in schools with lower levels, the difference in teachers’ trait EI becomes less important. Therefore, the positive relationship between teachers’ trait EI and job satisfaction is weaker in schools with higher levels of organizational trust than in those with lower levels. This echoes findings that trait EI is an asset as regards academic performance for vulnerable adolescents, but not so much for those who have strong cognitive skills (Petrides et al., 2004).

Last, the hypothesized relationship between principals’ trait EI and the job performance of teachers was not supported in this study. This stands in contrast with previous work that reported leaders’ trait EI positively influencing subordinates’ job performance (e.g., Wong and Law, 2002; Miao et al., 2018). Possibly, this deviation from earlier results may be due to the structure, size, and culture of Chinese primary schools; here, principals do not hold direct administrative roles in relation to every frontline teacher. Thus, the direct effects of leaders’ trait EI on subordinates’ job performance found in many business organizations may be much less discernible. Another plausible explanation for the difference in our results regarding the hypothesized relationship may be the small number of principals included in the study.

### Practical Implications

Our study has several practical implications regarding teachers’ job performance. First, it revealed that teachers’ trait EI can play an important role in promoting job performance in the workplace. Previous research has provided preliminary evidence that trait EI can be optimized through targeted training (e.g., Nelis et al., 2009, 2011). Thus, it is suggested that educational administrators and policymakers value its importance and incorporate it into training programs for primary school teachers. Although Chinese educators have increasingly recognized the value of trait EI and its suitability for improving educational practices (Chan, 2004), most efforts have been placed on the development of students’ trait EI, while training for school teachers remains insufficient (Yin et al., 2013). The need for EI training to achieve positive outcomes is especially important in mainland China, where teachers are faced with extensive educational reforms that put them under great performance pressure (Yin et al., 2014).

Since our findings suggest that higher levels of organizational trust in schools weaken the influence of teachers’ trait EI on job satisfaction and, in turn, on job performance, building organizational trust in schools may act as a protective factor for low trait EI teachers. School leaders and teachers should be encouraged to pay attention to the quality of trust relationships within their schools in order to strengthen job satisfaction and performance. School leaders could play a central role in promoting a climate of trust within schools (Liu et al., 2016). This is especially true in mainland China, as it is highly influenced by the dominant Confucian value of collectivism and the extended practice of vertical leadership (Tsui and Farh, 1997; Hawkins, 2000). Hence, a deliberate expression of care and support from principals becomes particularly important for shaping positive social relationships in Chinese school settings (Liu et al., 2016). As regards teachers, it seems advisable to embrace the idea that a trusting environment is a pathway to optimal teaching in their schools (Van Maele and Van Houtte, 2012). Thus, they should seek to engage in positive interactions with their colleagues that can foster a shared perception of trustworthy relationships.

### Limitations and Future Research

When interpreting our findings, several limitations should be borne in mind, which, at the same time, offer new avenues for research. First, the cross-sectional design of the study makes it difficult to determine the direction of causality between variables, which precludes the identification of any cause-effect relationships. As such, even though previous research supports the hypothesized directions of relationships, we encourage researchers to test our model in a longitudinal way to establish the underlying causal inferences with greater certainty.

Second, the sample was sourced from a limited number of schools of uneven size. This could result in an unfavorable influence on the studied cross-level effects. For example, the cross-level relationship between principals’ trait EI and teachers’ job performance could have turned out to have a larger effect if an even size of schools had been addressed or more schools had been approached. Moreover, all of the 37 sampled schools were located in the Hubei Province, China, restraining the generalizability of the findings. In summary, expanding the school samples in terms of numbers as well as areas would be a worthwhile approach for future studies.

A third limitation concerns the measurement methodology for several focal variables. Job performance was operationalized only through teachers’ self-reports, which may lead to social desirability bias. Although this method is consistent with prior research on the relationship between EI and job performance (Carmeli, 2003; Wu, 2011), we acknowledge that it may produce different results from other sources. Furthermore, several focal variables were measured from the same source at the same time, which could induce common method bias. However, it should be noted that the moderated mediation model is actually less likely to be detected when relationships are artificially inflated (Edwards and Lambert, 2007).

A fourth limitation is that our model was largely restricted to the positive influence of teachers’ trait EI on job performance, mediated by job satisfaction and moderated by organizational trust at the school level. There are likely to be other constructs that influence these relationships. For example, the results indicated that job satisfaction only partially mediated the relationship between teachers’ trait EI and job performance. In line with previous research, other attitudinal variables, such as organizational commitment (Anari, 2012), may also partly capture the complex processes underlying the link between teachers’ trait EI and job performance. Moreover, additional powerful contextual influences may well reside at various levels of analysis, such as workplace social support (Ju et al., 2015) or power distance (Miao et al., 2016). In line with our multilevel model, it would be important to identify other contextual variables that could enhance or diminish the effect of teachers’ trait EI on their job outcomes. In addition, leadership styles, which could account for the mechanisms by which principals’ trait EI affects teachers’ job performance, were not included in the current research design. Future studies could expand our findings by incorporating leadership styles into the proposed model.

## Conclusion

Our study contributes significantly to the literature in that it relates teachers’ trait EI to job performance and tests a multilevel model involving a moderated mediation relationship to reveal the underlying mechanisms. First, our findings confirmed that teachers’ trait EI positively influences their job performance directly and indirectly via job satisfaction at the individual level, thus supporting Hypotheses 1 and 2. Second, we demonstrated that school-level organizational trust could moderate the indirect effect from teachers’ trait EI to their job performance via job satisfaction, thus supporting Hypotheses 3 and 4. The multilevel model showed that the effect of teachers’ trait EI on job performance through job satisfaction was stronger in schools with lower levels of organizational trust. Third, no significant relationship between principals’ trait EI and teachers’ job performance was detected. Thus, Hypothesis 5 was not supported in the current study. Collectively, the various predictors were found to account for 15% of the variance in job performance. Consistent with our findings as well as previous work, it appears that teachers’ trait EI is indeed centrally important to the teaching performance, although its effects may vary across different settings. We look forward to future research that further examines the mechanisms underlying this important phenomenon.

## Author Contributions

ML and YM were involved in collecting the data. ML and PP-D developed the theoretical model, performed the data analyses, and drafted the manuscript with input from all authors. KP contributed in clarifying the theoretical model, revised the manuscript and provided feedback. KP and YM supervised the design and implementation of the project. All authors contributed to manuscript revision, read and approved the submitted version.

## Conflict of Interest Statement

The authors declare that the research was conducted in the absence of any commercial or financial relationships that could be construed as a potential conflict of interest.
